# Morning Plasma Melatonin Differences in Autism: Beyond the Impact of Pineal Gland Volume

**DOI:** 10.3389/fpsyt.2019.00011

**Published:** 2019-02-06

**Authors:** Anna Maruani, Guillaume Dumas, Anita Beggiato, Nicolas Traut, Hugo Peyre, Alicia Cohen-Freoua, Frédérique Amsellem, Monique Elmaleh, David Germanaud, Jean-Marie Launay, Thomas Bourgeron, Roberto Toro, Richard Delorme

**Affiliations:** ^1^Child and Adolescent Psychiatry Department, Robert Debré Hospital, Paris, France; ^2^Human Genetics and Cognitive Functions, Institut Pasteur, Paris, France; ^3^Pediatric Radiology Department, Robert Debré Hospital, Paris, France; ^4^Department of Pediatric Neurology, Robert Debré Hospital, AP-HP, Paris, France; ^5^Neuropaediatric Team, UNIACT, NeuroSpin, CEA-Saclay, Gif-sur-Yvette, France; ^6^Biochemistry Department, INSERM U942, Lariboisière Hospital, Assistance Publique-Hopitaux de Paris EA 3621, Paris, France

**Keywords:** pineal gland, MRI, children, sleep, circadian rhythm, autism spectrum disorder

## Abstract

While low plasma melatonin, a neuro-hormone synthesized in the pineal gland, has been frequently associated with autism, our understanding of the mechanisms behind it have remained unclear. In this exploratory study, we hypothesized that low melatonin levels in ASD could be linked to a decrease of the pineal gland volume (PGV). PGV estimates with magnetic resonance imaging (MRI) with a voxel-based volumetric measurement method and early morning plasma melatonin levels were evaluated for 215 participants, including 78 individuals with ASD, 90 unaffected relatives, and 47 controls. We first found that both early morning melatonin level and PGV were lower in patients compared to controls. We secondly built a linear model and observed that plasma melatonin was correlated to the group of the participant, but also to the PGV. To further understand the relationship between PGV and melatonin, we generated a normative model of the PGV relationship with melatonin level based on control participant data. We found an effect of PGV on normalized melatonin levels in ASD. Melatonin deficit appeared however more related to the group of the subject. Thus, melatonin variations in ASD could be mainly driven by melatonin pathway dysregulation.

## Introduction

Autism spectrum disorders (ASD) are a heterogeneous group of conditions characterized by a deficit in social communication and the presence of restrictive and repetitive behaviors and interest (DSM-5), affecting more than 1% of the general population (1, 2). Phenotypic heterogeneity exists because of differences in the degree of severity and due to the presence of frequent comorbid disorders associated with ASD such as intellectual disability (ID), attention-deficit hyperactivity disorder (ADHD) or sensory-motor disorders. One of the most frequent complains of individuals with ASD and their families is sleep disorders, ranging from 40 to 86% of the patients (3–6). The symptoms are very heterogeneous including increased rapid eye movement (REM) sleep latency, immature organization of rapid eye movements, decreased total sleep time or increased proportion of stage 1 sleep (7). Sleep disturbances significantly impact the daily functioning of patients, affecting their cognitive resources (mainly attention, flexibility, or working memory), but also altering their abilities to regulate their emotions and their behaviors (5).

Among the biological parameters involved in circadian rhythm, melatonin is one of the major regulators of the sleep cycle. In the general population, melatonin plasma levels are lower during the day and higher at night, with maximum peak secretion occurring typically 3–4 h after falling asleep (8). Besides its role as a circadian regulator, melatonin also acts as a pleiotropic molecule, showing antioxidant, immunomodulatory, anti-inflammatory, and anti-coagulopathic properties (9). Melatonin also modulates neuronal networks by changing the circadian neuronal transmission via direct effect of MT1 and MT2 synaptic receptors (10–14). Melatonin also participates with the clock neurons to the generation of circadian oscillations in neuronal transmission (15). In ASD, low plasma melatonin concentration (or of its urinary metabolites) was recurrently reported (3, 16). For example, our group showed a low level of plasma melatonin (below 0.07 nM at 8.00 a.m.) in 51% of individuals with ASD and in ~25% of their unaffected relatives (with a similar prevalence in the parents and in their unaffected siblings) (3). This dysfunction of the melatonin pathway might account for the high prevalence of sleep difficulties in patients with ASD as well as their parents and their relatives (17, 18) Several studies evaluated the effect of night-time administration of melatonin. One of them showed that supplemental melatonin improved sleep latency (measured by actigraphy) and sleep behavior in most children at doses of 1 or 3 mg. It was effective in week 1 of treatment, maintained effects over several months, was well-tolerated and safe, and showed changes in sleep latency which decreased significantly (19). Moreover, melatonin supplementation concurrent with behavioral interventions (including parental educations) was the most effective strategy to improve the sleep difficulties in patients with ASD (20). The administration of melatonin (specifically of prolonged-release melatonin) allowed an increase of the total sleep time (approximately of 1 h/night) and a decrease in sleep latency (with a mean decrease of a half hour) without causing earlier wakeup time. Subjects who received melatonin also reported a global decrease of overall sleep disturbance (21). Although low plasma melatonin levels appeared to be a robust trait in a subgroup of individuals with ASD, the underlying biological mechanisms remained largely unknown (3, 22).

Melatonin derives from serotonin, which is successively converted into N-acetylserotonin (NAS) and melatonin by the enzymes arylalkylamine N-acetyltransferase (AANAT, EC: 2.3.1.87) and acetylserotonin O-methyltransferase (ASMT, EC: 2.1.1.4) (23). We identified several rare mutations of ASMT and recently suggested that post-translational and post-transcriptional mechanisms may affect the synthesis of melatonin. We also observed reduced levels of 14-3-3 proteins, which regulate AANAT and ASMT activities, and increased levels of miR-451, targeting 14-3-3ζ (24). However, additional factors, such as an abnormality of the pineal gland development itself have not been considered to date.

Melatonin is synthesized by the pinealocytes in the pineal gland, a small conical endocrine gland of about 100 mm^3^, located medially in the vertebrate brain (25). Several studies showed a correlation between the pineal gland volume (PGV) and the plasma melatonin concentration in the general population (25–27) but also in patients with severe pineal gland hypoplasia linked to mutations in the transcription factor *paired box 6 PAX6 gene* and associated with sleep impairments (28). There is considerable heterogeneity in the literature on variations in brain volume or subcortical structures in ASDs (29). Particularly, the topologic alterations in brain structure in patients with sleep disorders remain largely unknown (30) and only a few studies have focused on the morphology of the pineal gland itself. One study investigated the morphology of the pineal gland in healthy volunteers and concluded that uncalcified solid pineal tissue is related to human saliva melatonin levels, and suggested a linkage between better sleep quality and hormonal active pineal tissue (31). However, to date, volumetric abnormalities of the pineal gland have not been considered to explain melatonin levels in ASDs. The aim of this study was thus to investigate the correlation between PGV and plasma melatonin in patients with ASD, their first-degree unaffected relatives (since melatonin levels and brain volume are highly heritable conditions) (32, 33) and in controls from the general population. Based on previous findings, we hypothesized that the low melatonin levels observed in ASD could be related to a decreased volume of the pineal gland.

## Methods

### Sample

A sample of 219 individuals composed of 81 with ASD and their 90 unaffected relatives were compared to 48 control participants (Table 1). Participants with ASD were from the C0733 cohort and recruited at the Child and Adolescent Psychiatry Department, Robert Debré Hospital, Paris (France). Patients with ASD were included after a systematic clinical and medical evaluation including negative testing for Fragile-X syndrome. Diagnosis of ASD was based on DSM-IV TR criteria and made by adding the information from the Autism Diagnosis Interview-Revised (ADI-R) (34), the Autism Diagnostic Observation Scale (ADOS) (35), and clinical reports from experts in the field. Intellectual functioning of all participants was estimated with the Raven's Progressive Matrices or with the Wechsler Intelligence Scales. Relatives were included after the same check-up and had no ASD. Participants from the control group were from the general population. All subjects from the control group with a personal or a familial history of ASD were not included. Control participants had no academic difficulties, were enrolled in a regular school level and had never had any speech therapy, psychiatric or neurological diagnoses or traumatic brain injury, severe prematurity (<1,850 g at birth), or epilepsy. Participants were classified by “group” (as patients, relatives, or controls) for analysis. Four individuals were excluded from the final analysis: two due to our inability to properly estimate the PGV and two participants were excluded since they displayed pineal cysts over the 95th percentile (see the results for more details).

**Table 1:** Demographic characteristics of the participants enrolled in the study.

	**ASD**	**Relatives**	**TD**	***P*-value**
Number of participants	78	90	47	
Males (%)	60 (76)	45 (50)	25 (53)	9.86 × 10^−5^
Age (*SD*)	13.2 (8.8)	35.7 (17.5)	22.0 (12.7)	2.51 × 10^−20^
Non-verbal IQ (*SD*)	95.3 (26.1)	115 (12.8)	111.2 (16)	8.31 × 10^−8^
SRS (T-score)	77.56 (11.54)	46.55 (8.98)	47.46 (7.37)	1 × 10^−18^
**ADI-R**				
ADI-R—Social (*SD*)	18.46 (8.9)			
Communication (*SD*)	8.22 (4.9)			
Repetitive behaviors (*SD*)	6.01 (3.5)			
**ADOS**				
Communication/Social (*SD*)	15.62 (0.59)			
Repetitive behaviors (*SD*)	2.16 (0.25)			

*For continuous variables, data are mean ± standard deviation (SD); ASD, Autism Spectrum Disorder; TD, Typically Developing Children; N, Number of Participants; y, Age in Year at the Inclusion. ADI-R, Autism Diagnostic Interview Revised; ADOS, Autism Diagnostic Observation Schedule; ASD, Autism Spectrum Disorder; r; IQ, Intellectual Quotient; SD, Standard Derivation; SRS, Social Responsiveness Scale*.

### Ethics Statement

This study was carried out in accordance with the recommendations of the local ethics committee of Hospital Robert Debré with written informed consent from all participants. All subjects gave written informed consent in accordance with the Declaration of Helsinki. The protocol was approved by the Local Ethics Committee (study approval no. 08-029).

### Pineal Gland Volume Estimation Based on Magnetic Resonance Imaging

For all participants, MRI data were collected using parameters that were described previously, with a 1.5 Tesla scanner using a T1-Weighted acquisition (36).

Raw DICOM images were converted to NIFTI format with dcm2niix (https://github.com/rordenlab/dcm2niix) and defaced with MRIdeface (37). To estimate brain volume, we extracted the brain by registering it to the MNI152 atlas and automatically segmented gray and white matter tissues with the tool FSL FAST v5.0.10, following the fsl_anat pipeline (https://fsl.fmrib.ox.ac.uk/fsl/fslwiki/fsl_anat) (38–40). We used BrainBox, an open access software, to visually control the quality of the images (http://brainbox.pasteur.fr/). When automatic and manual segmentations did not allow the estimation of the total brain volume and/or the pineal gland volume, the subjects were excluded from the study (*n* = 2). To estimate the total volume of the pineal gland, we performed a manual segmentation following the method described previously by Sarrazin et al. (41). We used the posterior part of the third ventricle, the quadrigeminal cistern, the corpus callosum, and the upper colliculus as anatomical landmarks to delineate the PGV. Since cysts are frequently reported ranging from 1.5 to 10.8% in the general population (42), we systematically screened the images to reduce the risk of overestimation of the total PGV. Two individuals (*n* = 1 ASD; *n* = 1 control) with large pineal gland cysts and a PGV over the 99th percentile were excluded from the study.

### Blood Sampling and Plasma Melatonin Measurement

The plasma melatonin measurements for each participant included in this study have been reported previously by Pagan et al. (3). Only participants with MRI data (allowing the estimation of the PGV) were included in this study. Briefly, all participants were free of any treatment interfering with melatonin synthesis and had to avoid any food with a high monoamine content such as chocolate, bananas or nuts, during 2 days before blood sampling. Samples were collected between 8:30 and 10:30 a.m. using ACD-A tubes. Plasma melatonin was measured using a radioimmunoassay (RK-MEL, Bühlmann, Switzerland) according to the manufacturer's instructions.

### Statistical Analyses

Descriptive statistics were first performed using the nonparametric Mann–Whitney–Wilcoxon test to analyze difference between groups. Secondly, multiple linear regression models were applied to study the relationship between PGV and the plasma melatonin. We next generated a normative model of plasma melatonin using a non-parametric LOESS approach (43). All statistical data analyses were performed using the JMP Pro Version 10.0.2 software (http://www.jmp.com), Python 2.7 (numpy 1.10.2 and scipy 1.0.0), and G ^*^ Power v3.1 (http://www.gpower.hhu.de).

The original version of the non-parametric normative modeling used Gaussian Processes (GP) to model the distribution of control group measures (43). We calculated a data-driven normative model with a non-parametric adaptation using LOESS (LOcally wEighted Scatter-plot Smoother; mathematical method to approximate a distribution by locally fitting a polynomial to data) (44). The resulting normative score combined the empirical mean and standard deviation deduced from LOESS on the PGV of the control participants. It can thus be understood as a Z-score quantifying the deviation of each subject from the predicted value based on the normative model.

(https://github.com/GHFC/SoNeTAA/blob/master/Retrospective/NPNM.py).

## Results

### Plasma Melatonin and Pineal Gland Volume

**Figure 1:**
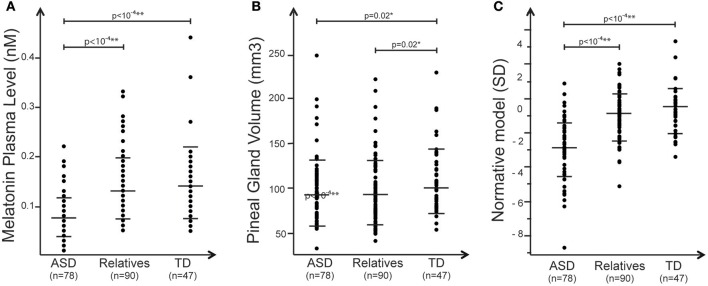
Summary of the main effects observed in the study. Plots of **(A)** melatonin (nM) measured for each group **(B)**, pineal gland volume distribution (mm^3^), and **(C)** melatonin normative model of melatonin corrected for PGV. In all panels, black dots represent the individual data, and horizontal long and short bars are, respectively, mean and standard deviations.

Melatonin plasma level was assessed in the early morning in individuals with ASD, their first-degree unaffected relatives and in a sample of typically developing individuals. With the nonparametric test we observed a significant association between blood melatonin and the group of the participants enrolled in the study (*R*^2^ = 0. 21; *p* = 1.3 × 10^−11^) (Table 2). Analysis revealed that patients displayed significantly lower melatonin levels than controls and unaffected relatives (respectively, *z* = 6.2, *p* = 4.10^10^; *z* = 6.8 *p* = 1.10^−11^). Relatives displayed to a lesser extent a reduced level of melatonin compared to controls, but that did not reach significance *z* = 0.88, *p* = 0.38) (Figure 1). We then estimated the volume of the pineal gland based on 3DT1 brain MRI, by using a manual segmentation of the pineal gland. Analysis revealed that the volume of the pineal gland was lower in patients than in controls, and lower in relatives than in controls (respectively, *z* = 2.21, *p* = 0.02; *z* = 2.28, *p* = 0.02) (Figure 1). We did not observe in our sample any correlation between the volume of the pineal gland and the total brain volume (*R*^2^ = 0.8, *p* = 0.25) or age (*R*^2^ = −0.08, *p* = 0.22 or the severity of social communication deficit (as measured with the SRS total t-score) (*R*^2^ ≤ 0.001).

**Table 2:** Plasma melatonin level and brain volumes for patients with Autism spectrum disorder (ASD), their first-degree relatives, and typically developing children (TD).

	**ASD**	**Relatives**	**TD**	***R*^**2**^ (*p*)**
Plasma morning melatonin level (nM)	0.08 (0.04)	0.13 (0.06)	0.14 (0.07)	0.21(1.3 × 10^−11^)
Pineal gland volume (mm^3^)	96.1 (36.7)	96.2 (35.7)	108.8 (35.5)	0.02 (0.10)
Total brain volume (cm^3^)	1169.1 (123.3)	1135.7 (119.6)	1152.7 (88.0)	0.02 (0.17)

*Data are mean ± standard deviation (SD)*.

### Interactions Between Pineal Gland Volume and Blood Melatonin

**Figure 2:**
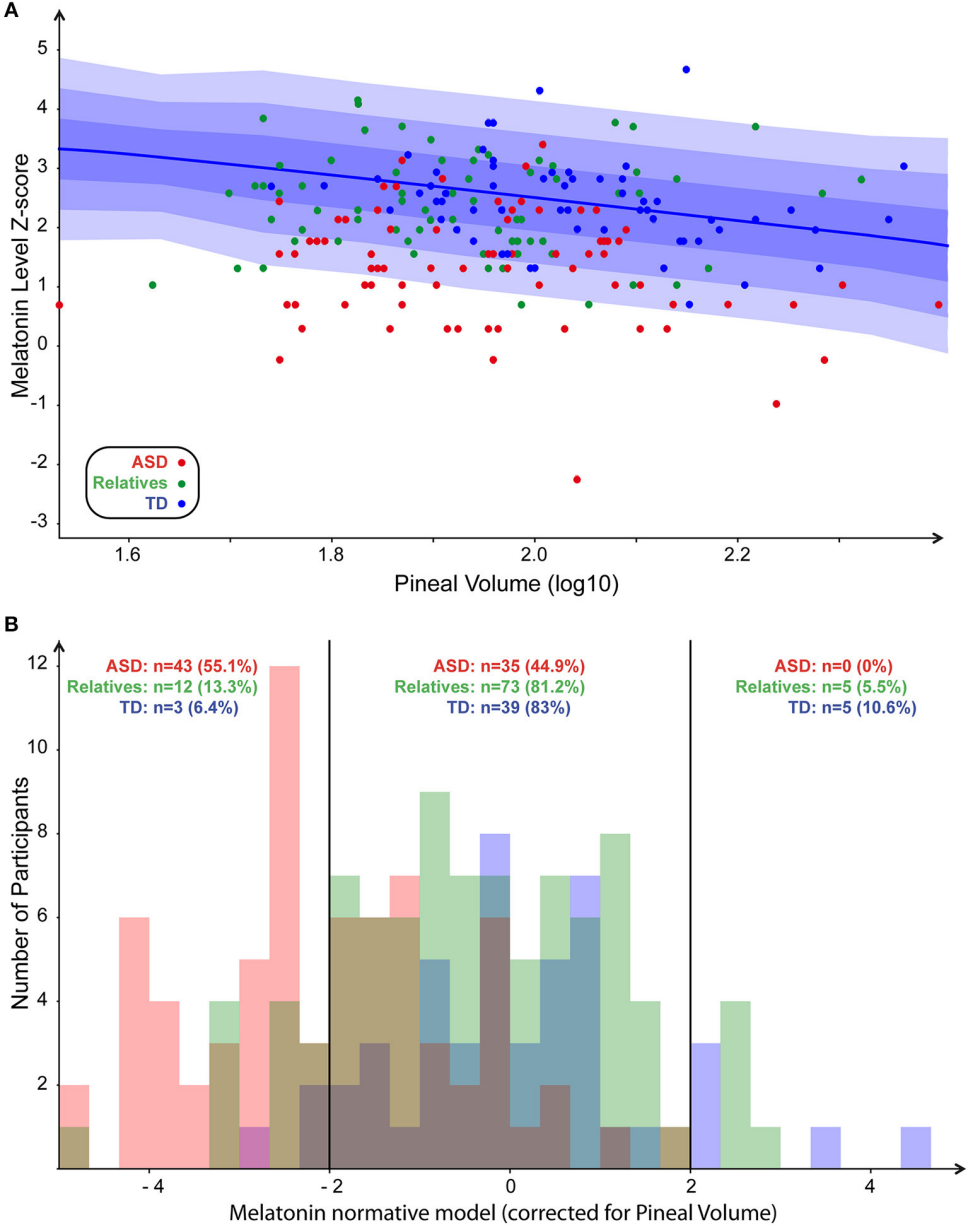
Normative model of melatonin. **(A)** Scatter plot of melatonin levels in function of PGV (both values log normalized) with normative model overlaid (1, 2, and 3 SD, respectively, for dark, medium, and light blue). **(B)** Distribution of the number of participants in each group around the normalized pineal volume. Participants with ASD, first-degree relatives, and controls are in red, green, and blue, respectively.

To explore the correlation between blood melatonin levels and the PGV, we built a linear model including not only the PGV but also the demographic or the clinical variables, which could act as confounding factors. Age, sex, IQ, group of the participants, total brain volume, and their interactions were included in the final analysis. The resulting regression appeared to have a statistically significant predictive capability [*F*_(27, 144)_ = 4, *p* = 1 × 10^−4^], with *R*^2^ = 0.43. More specifically, we observed that blood melatonin was affected by the group of the participants [*F*_(27, 144)_ = 9.27; *p* = 0.0002] but also by the PGV [*F*_(27, 144)_ = 5.17; *p* = 0.02] and the interaction between the group and IQ [*F*_(27, 144)_ = 3.6; *p* = 0.02]. While there was an effect of total volume on pineal volume (*R*^2^ = 0. 99; *p* < 0.0001), the melatonin level was not related to total brain volume [*F*_(27, 144)_ = 0.18, *p* = 0.67]. To further understand the interaction between melatonin, PGV and the group of the participant, we generated a normative model exploring the melatonin levels in the three groups of participants, corrected for the effect of the PGV as seen in controls. We log-normalized all the values, fit the normative model on controls (Figure 2A), and then quantified for every participant how much melatonin deviated from the normative model, i.e., from typically developing participants with a similar PGV. The normative score thus gave a measure comparable to a Z-score corrected for normal effect of the PGV. We then extracted the percentages of participants with an extreme normative score (<-2SD or >+2SD) in patients, their first relatives, and controls. We found that 55% of the participants with ASD had low melatonin levels corrected for PGV (<-2SD) while none of them had high levels (>+2SD) (Figure 2B). *Post-hoc* analysis based on the normative model (i.e., after correction of PGV effect) confirmed that patients displayed significantly lower melatonin levels than controls (*z* = 6.9, *p* < 10^−4^) and relatives showed a moderate but not significant deficit compared to controls (*z* = 1.4, *p* = 0.16). Despite this lack of correlation between the PGV and the total brain volume or age, we replicated our analysis by controlling for both age and brain volume using either a linear model or a normative model. The two complementary analyses support that age effects did not account for differences in melatonin levels between groups. Based on this analysis, we extracted the percentages of participants with extreme normative scores (i.e., below −2SD). We found in a similar way that 23% of the participants with ASD had low melatonin levels corrected for PGV (below −2SD) when considering the linear correction and 50% for non-linear correction (see Supplementary Figures S1, S2). To ensure the reliability of our results given our population, we quantified the statistical effect size of the normative model. With an estimated effect size of *d* = −1.46 (α = 0.05), 11 patients would have been enough to reach a statistical power of 80%.

## Discussion

The goal of our study was to further assess the factors that could result in low blood melatonin levels in autism. The mechanisms underlying such abnormality remained unclear although significant effort has been made, specifically in the identification of deleterious mutations affecting the melatonin pathway (22, 23, 33). Surprisingly, no study explored whether the observed melatonin deficiency could result from a direct decreased volume of the pineal gland, i.e., a putative decreased number of pinealocytes. Using a voxel-based volumetric measurement method, we made accurate measurements of the PGV and examined its correlation with melatonin. We observed that morning blood melatonin level was mainly associated with the group of the participants and to a lesser extent, but not significantly, with the PGV. Our normative modeling confirmed that the effect of the PGV on melatonin was weak, fostering the hypothesis of a melatonin pathway primary or secondary deficit in autism.

Our sample of patients, relatives, and controls were representative of the larger sample enrolled in Pagan et al.'s study (3). The low melatonin levels were detected in the probands and to a lesser extent in their relatives (45, 46). These results were in accordance with the decrease of the 6-sulfatoxymelatonin (one of the urinary melatonin metabolite) levels reported in independent samples of individuals with ASD (16) and in mothers with a child with ASD (47). We previously showed that heritability of melatonin levels in families with ASD was ~0.2 (33, 48). Interestingly, the melatonin heritability estimate was higher for unaffected relatives (*h*^2^ > 0.4) than for children with ASD (approximately *h*^2^ = 0.1), suggesting that environmental factors may affect the melatonin pathway in the context of autism.

In our study, we hypothesized that low melatonin could be correlated to PGV in participants with ASD. Even if we correlated to the status but not to the dimensional aspect of the severity, we observed a smaller PGV very similar in participants with ASD and in their first-degree relatives compared with controls. The volume was significantly lower in individuals with ASD compared to controls, corresponding to an average drop of 12.6 mm^3^ (Table 2). Our results were in accordance with those reported in schizophrenia (49, 50) but differed from reports in major depression or bipolar disorder (41, 50), in which no differences have been found compared to controls. The lessening of the PGV may be related to a “broad phenotype” of a social communication deficit, since not only patients with ASD and their first first-degree relatives shared a load of social impairments (51, 52) but also subjects with schizophrenia (53, 54). In parallel, small PGV has also been reported in subjects with primary insomnia (27) or sleep-rhythm disturbance (31). Thus, the abnormalities of PGV we observed in patients with ASD and their relatives might be related to the sleep abnormalities frequently reported in these subjects (55, 56). Indeed, in the literature, 40–80% of the subjects with ASD showed a wide range of sleep difficulties, and for half of them bedtime resistance problems, insomnia, parasomnias, and problems waking in the morning (57). Finally, the lessening of the PGV in ASD subjects and their relatives was not associated with any similar reduction of total brain volume. This was also supported by additional studies in which the total intracranial volume was not correlated with the PGV (41, 58). This suggests that the biological and environmental factors, which contributed to the PGV, were independent from those involved in whole brain growth. By building a linear model that integrated various clinical covariates (sex, age, IQ, and the group of the participants), we observed that plasma melatonin appeared more related to the group of the participants than to the PGV.

Using the normative model, we further tried to delineate the complex relationship between the melatonin pathway, the group of the participants, and additionally the PGV, because within this subgroup melatonin seemed to be negatively correlated to the PGV (Figure 2B). The normative approach can better account for phenotypic heterogeneity than traditional case-control models because of the ability to isolate individuals expressing significant PGV-deviance. By normalizing the effect of PGV on melatonin, we indeed observed a major skewed distribution of melatonin in patients with ASD (since 55% of them displayed melatonin levels more than two standard deviations below the expected value based on the normative model). Our results corroborated those reported in the literature. For example, in a large sample of healthy participants (*n* = 113), the PGV correlated with saliva melatonin (31), suggesting that PGV had a major impact and that additional factors regulated the melatonin pathway.

Our study has some limitations. The weak correlation between melatonin level and the PGV may also result from the method we used to measure melatonin. One study showed that the correlation was stronger when considered the maximum of the circadian melatonin concentration or the 24-h melatonin secretion (*r* = 0.61 and *r* = 0.64, *p* < 0.05), but not its minimum (26). In our study, one of the major limitations is that we assessed melatonin only from plasma sampled in the morning despite the fact that melatonin displays marked nychthemeral variations, with a peak occurring at night (59). The estimation of melatonin level based on morning dosage probably limits our findings. However, many studies have reported a high degree of correlation between nocturnal measurements of urinary aMT6s and plasma and serum melatonin (60). We measured melatonin levels between 8 and 9 a.m. During this period, melatonin levels were still relatively high, with minimum levels reached during the afternoon (61). Our results are therefore probably not so much impacted by the sampling.

Our study was also limited in its ability to precisely quantify the PGV. The MPRAGE sequences were not specifically designed to perform this study. The investigation of the morphologically atypical gland would have required clearly defined sequences such as 3D fast imaging with steady state acquisition (FIESTA) sequences to enhance cyst detection (27, 62) as well as susceptibility-weighted imaging (SWI) sequences to quantify the level of calcification (31). The lack of detection of these gland abnormalities—specifically at their early stage—could alter the estimation of the PGV. For example, in a large cohort of healthy volunteers (*n* = 103) using the optimal sequences mentioned below, the estimated cyst volume accounted for ¾ of the pineal volume and the estimated calcification volume for ~10% (31). The presence of cysts and calcification increased along the lifespan and were frequently detected in older people (respectively, 59 and 21%) (63). Thus, in our study, calcification might have impacted the PGV of the controls since their ages were higher than those of participants with ASD (Table 1). We observed however that age effects did not account for differences in PGV between groups, which weakens the potential effect of age-related calcifications in our study. Finally, the non-detection of cysts and calcification may also explain the inhomogeneity of the PGV reported in the control populations across studies. The PGV estimates in healthy volunteers ranged from 71 mm^3^ (*n* = 86) by using MPRAGE sequences (27) to 155 mm^3^ (*n* = 103) by using FIESTA & SWI sequences (31). In our study the mean PGV estimated in controls (# 109 mm^3^) was very similar to the one reported by Sarrazin et al. (41) or after correction by Liebrich et al. (31).

In conclusion, even if a few studies showed a correlation between PGV and plasma melatonin concentration in the general population (26, 27, 41), no information was available about the link between PGV and melatonin in ASD. Our study suggests that PGV acted as a modest contributor to the melatonin deficit observed in ASD. Melatonin pathway dysregulation in ASD seems more related to post-translational and post-transcriptional mechanisms (24). For example, we recently showed a reduced level of 14-3-3 proteins in ASD. These proteins regulated AANAT and ASMT activities, the two enzymes involved in the melatonin synthesis. Further explorations combining clinical, biochemistry, and genetics in ASD may provide new avenues in the field.

## Author Contributions

AM and RD designed the study. AM, FA, AB, AC-F, ME, DG, J-ML, and RD contributed to data acquisition. AM, GD, HP, TB, RT, NT, and RD participated in data analysis and interpretation. AM and RD drafted the manuscript. All authors contributed to the critical revision and approved the manuscript.

### Conflict of Interest Statement

The authors declare that the research was conducted in the absence of any commercial or financial relationships that could be construed as a potential conflict of interest.

## Supplementary Material

Supplementary Figure S1 Normative model of melatonin with linear correction of the pineal gland volume for both age and total brain volume. **(A)** Scatter plot of melatonin levels in function of corrected PGV (both values log normalized) with normative model overlaid (1, 2, and 3 SD, respectively for dark, medium and light blue). **(B)** Distribution of the number of participants in each group around the normalized pineal volume. Participants with ASD, first-degree relatives, and controls are respectively in red, green, and blue.

Supplementary Figure S2 Normative model of melatonin with non-linear correction of the pineal gland volume for both age and total brain volume. **(A)** Scatter plot of melatonin levels in function of PGV (both values log normalized) with normative model overlaid (1, 2, and 3 SD, respectively for dark, medium and light blue). **(B)** Distribution of the number of participants in each group around the normalized pineal volume. Participants with ASD, first-degree relatives, and controls are respectively in red, green, and blue.

## Abbreviations

ADHD: attention-deficit hyperactivity disorder
ASD: Autism spectrum disorder
FIESTA: fast imaging with steady state acquisition
ID: intellectual disability
IQ: intellectual quotient
PGV: Pineal Gland Volume
TD: typically developing children.
