# Supplementary figures and images for: Morning Plasma Melatonin Differences in Autism: Beyond the Impact of Pineal Gland Volume
Source: Front Psychiatry. 2019 Feb 6;10:11. doi: 10.3389/fpsyt.2019.00011 (PMC6372551; doi:10.3389/fpsyt.2019.00011)

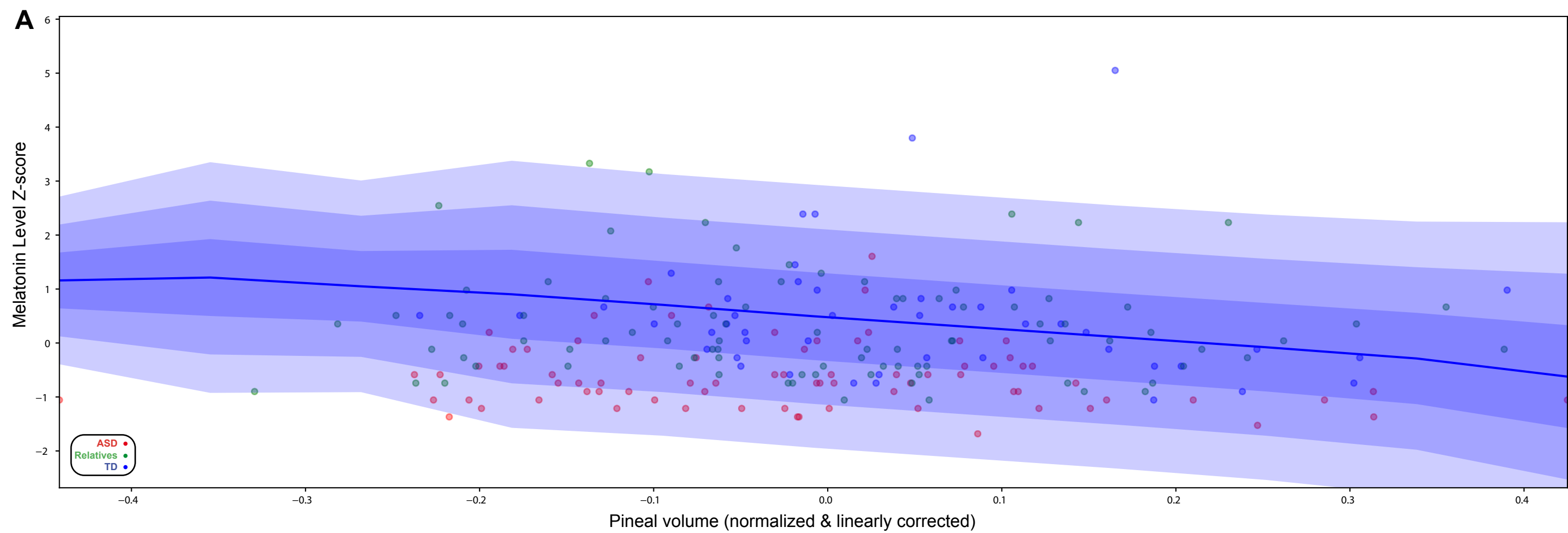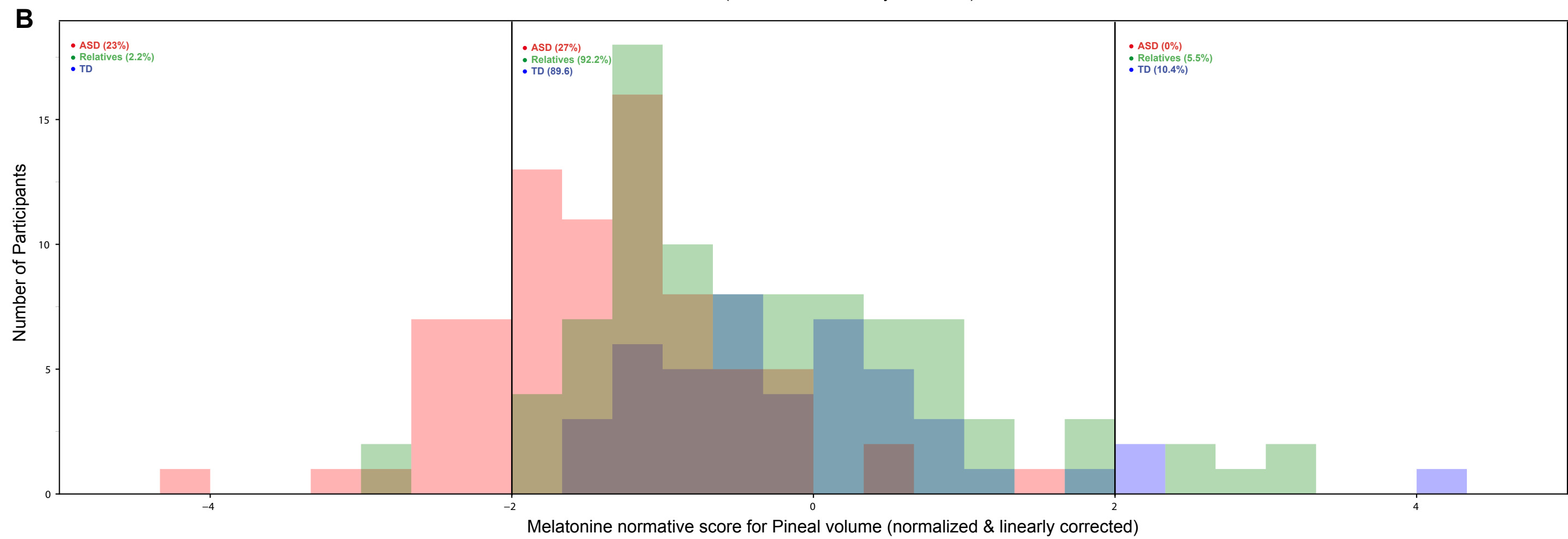

Supplement: Supplementary Figure S1 — Normative model of melatonin with linear correction of the pineal gland volume for both age and total brain volume. (A) Scatter plot of melatonin levels in function of corrected PGV (both values log normalized) with normative model overlaid (1, 2, and 3 SD, respectively for dark, medium and light blue). (B) Distribution of the number of participants in each group around the normalized pineal volume. Participants with ASD, first-degree relatives, and controls are respectively in red, green, and blue. [file Image_1.pdf]

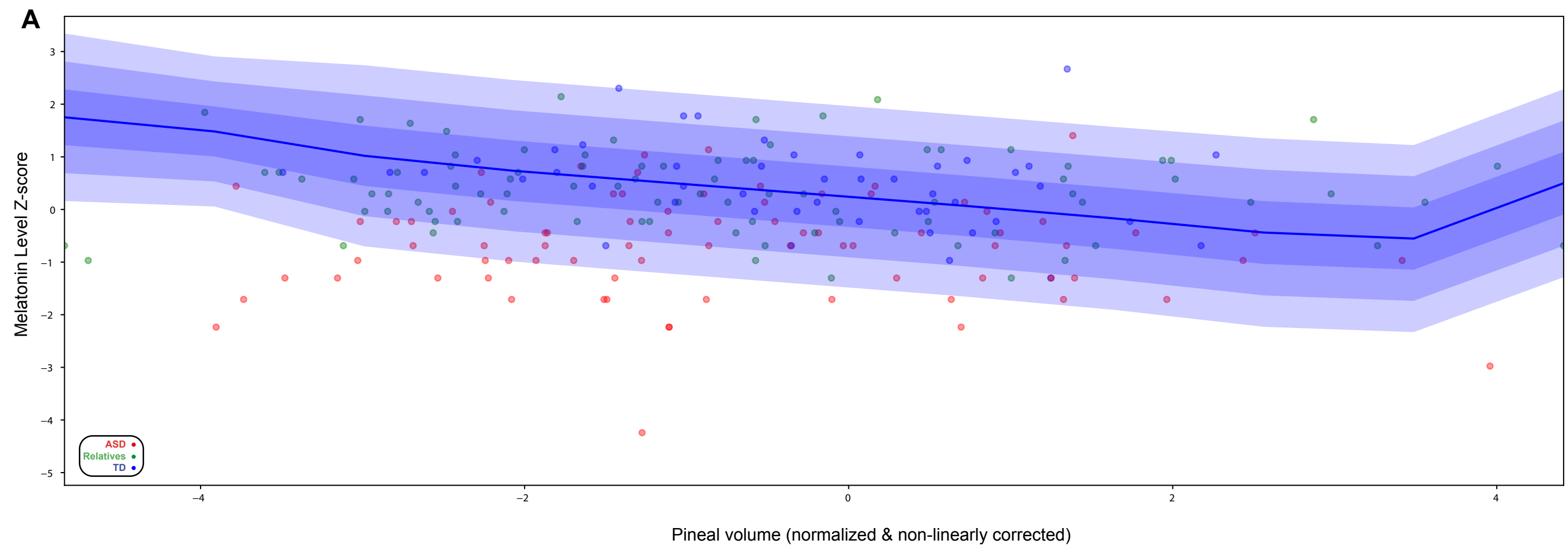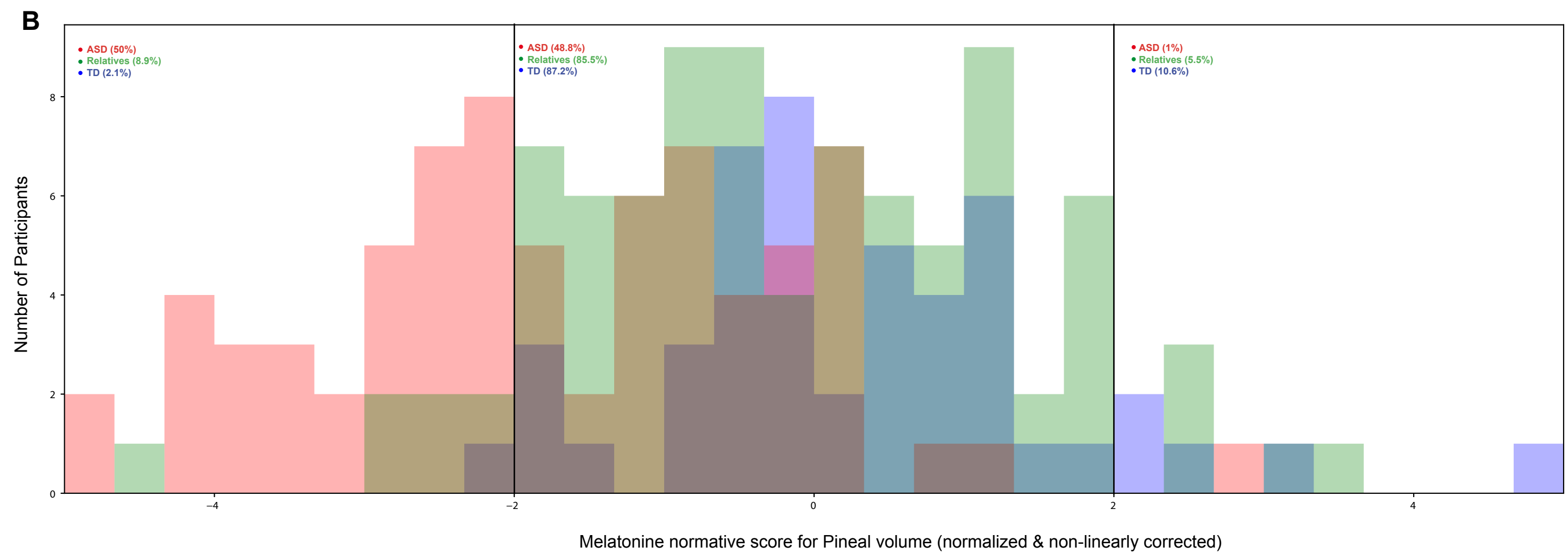

Supplement: Supplementary Figure S2 — Normative model of melatonin with non-linear correction of the pineal gland volume for both age and total brain volume. (A) Scatter plot of melatonin levels in function of PGV (both values log normalized) with normative model overlaid (1, 2, and 3 SD, respectively for dark, medium and light blue). (B) Distribution of the number of participants in each group around the normalized pineal volume. Participants with ASD, first-degree relatives, and controls are respectively in red, green, and blue. [file Image_2.pdf]
